# The role of particulate matters on methylation of *IFN-γ* and *IL-4* promoter genes in pediatric allergic rhinitis

**DOI:** 10.18632/oncotarget.24227

**Published:** 2018-01-13

**Authors:** Youjin Li, Zhe Mu, Hongyang Wang, Jinfen Liu, Fan Jiang

**Affiliations:** ^1^ Department of Otorhinolaryngology-Head and Neck Surgery, Children's Medical Center, Shanghai Jiaotong University, Shanghai 200127, China; ^2^ School of Public Health, Shanghai University of Traditional Chinese Medicine, Shanghai 201203, China; ^3^ Shanghai Key Laboratory of Meteorology and Health, Shanghai Meteorological Bureau, Shanghai 200030, China; ^4^ Chinese PLA Institute of Otolaryngology, Chinese PLA General Hospital, Medical School of Chinese PLA, Beijing 100853, China; ^5^ Department of Pediatrics, Children's Medical Center, Shanghai Jiaotong University, Shanghai Jiaotong University Pediatric Institute, Shanghai 200127, China; ^6^ Department of Child Development and Behavior, Children's Medical Center, Shanghai Jiaotong University, Shanghai 200127, China; ^7^ MOE-Shanghai Key Laboratory of Children's Environmental Health, Shanghai 200127, China

**Keywords:** pediatric allergic rhinitis, particulate matter, methylation, IFN-γ, IL-4

## Abstract

Allergic rhinitis (AR) is a chronic inflammatory disorder driven by T cell activation. How particulate matter contributes to epigenetic changes that in turn influence cytokine gene expression in CD4^+^T cells remains unclear. In this study, 105 children diagnosed with AR and 90 healthy controls were recruited to explore the possible mechanism of particulate matter (PM) on the epigenetic regulation of CD4^+^T *IFN-γ* and *IL-4* promoter genes. Daily average PM_10_ and PM_2.5_ were obtained from five state-controlled monitoring stations, and activity-based dynamic exposure and personal exposure data were collected. DNA methylation patterns of *IFN-γ* and *IL-4* promoter regions were analyzed using bisulfite sequencing. mRNA levels were detected by real-time quantitative reverse transcription polymerase chain reaction. We found that the methylation rate in *IFN-γ* was higher in AR CD4^+^T cells than in the controls. *IFN-γ* mRNA expression was significantly decreased in CD4^+^T cells, and negatively correlated with the mean methylation level of *IFN-γ*. However, no correlation between *IL-4* methylation and *IL-4* mRNA expression was found. After adjusting for age, gender, exclusive breastfeeding within 4 months after birth and parental history of allergic disease, out data showed that PM_2.5_ exposure level was positively correlated with methylation level in *IFN-γ* promoter region and decreased cytokine expression. We conclude that the effect of PM_2.5_ on pediatric AR may be mediated through epigenetic modification of *IFN-γ* promoter region.

## INTRODUCTION

The prevalence of allergic diseases, such as allergic rhinitis (AR) in children appears to be increasing globally, including in China. In 2013, Zhang *et al.* reported an AR prevalence of 48% in 3–5 years old children in Beijing, China based on surveys via questionnaires [1]. Although the etiology of AR remains uncertain, there is evidence suggesting that the allergic response is associated with certain ambient air pollutant exposure [2].

Ambient particulate matter (PM) pollution is one of the most serious environmental challenges in China, with an important impact on human health, especially in children and other susceptible populations. The adverse health effects associated with air pollution exposure may be attributed to short-term (a few minutes to one day) or long-term (a few months to a few decades) exposure, and different pollutants may have widely different exposure-response characteristics [3]. Among pollutants, PM with an aerodynamic diameter less than 2.5 µm (PM_2.5_), as the primary air pollutant in China, is much higher than the recommended criterion by the WHO “global air quality guidelines” in 2006 [4]. PM2.5 can be easily conjugated to toxic heavy metals, organic pollutants, bacteria, viruses and other common allergens in the air, which causes far more damage to human health than PM_10_ (an aerodynamic diameter of less than 10 µm) due to its size and relatively large surface area [5]. A number of epidemiological studies have found that exposure to air pollution could generate a variety of negative health effects, including increased frequency and severity of AR, especially AR combined with asthma [6, 7].

Environmental pollutants play an important role in regulating the occurrence and progression of allergic diseases that may be regulated through genetic and epigenetic mechanisms. Epigenetic change in DNA methylation is recognized as an important transcriptional regulator, which allows the methylation of the promoter region and other regulatory sequences and tends to reversibly suppress gene transcription [8]. Sofer *et al* [9] reported that methylation patterns of asthma pathway were related to black carbon and sulfide exposures in the environment, suggesting that air pollution might affect airway response by changing gene methylation status.

The mechanism of chronic allergic inflammation is dominated by type 2 helper T cells (Th2) response, which mostly results from the imbalance between type I helper T cells (Th1) and Th2 that belong to CD4^+^ helper T cells. T helper cell polarization to Th2 usually changes the sections of Th1/Th2 signatory cytokines as shown in differentiation with IFN-γ and IL-4 [10, 11]. IFN-γ and IL-4 are among methylation sensitive genes given a large number of CpG islands and loci in the promoter regions of these genes, which can be regulated by methylation and play major roles in the process of epigenetic regulation [12]. In a murine model, PM is capable of changing DNA methylation status in peripheral blood of sensitized individuals as well as IFN-γ hypermethylation and IL-4 hypomethylation in CD4^+^ T cells, resulting in Th1/Th2 imbalance and cytokine expression change [13]. A hypermethylation with increased or hypomethylation with reduced level of CpG island is methylation relative to the normal tissue specific pattern. Therefore, environmental pollutants might lead to the occurrence of allergic diseases by altering methylation in the promoter regions of IFN-γ or IL-4, and aggravating Th1/Th2 imbalance. This finding would suggest a new paradigm for AR etiology.

As AR results from genetic and environmental conditions, high risk environmental factors on epigenetic changes can induce different cascade that contribute to individual cell recognition. Herein, we conducted a case-control study to demonstrate the dynamic exposure of PM on AR. The study was conducted in a group of children with AR with one-year dynamic exposure to PM_2.5_ and PM_10_. The Kriging interpolation method was used according to PM_10_ and PM_2.5_ data in combination with residential addresses and latitude and longitude data of meteorological monitoring sites. In addition, the Shanghai particle penetration coefficient model was applied as well as indoor and outdoor activity time [14]. The possible epigenetic mechanism of PM exposure in pediatric AR was then explored in combination with selected CpG methylation patterns of IL-4 and IFN-γ promoter genes in CD4^+^cells.

## RESULTS

### Subject demographic data

AR group was classified according to the serum allergen-specific IgE category. Serum IgE detection showed that allergens were 85 (80.6%), 25 (23.8%), 16 (80.6%) and 9 (15.2%) corresponding to house dust mites, tree powder, dog/cat hair and mixed allergens, respectively. The assessment of individual exposure to PM and blood sample collection at the end of the end of 2013 was performed in 105 children with AR and 90 matched cases recruited into the control group. General information and family demographic data are shown in Table 1. There were no differences between the two groups in most characteristics, except that it is significant different in the exclusive breastfeeding within 4 months after birth and parental history of allergic disease. In addition, the daily air pollution concentrations of PM_10_ and PM_2.5_ were 67.4 and 33.4 ug/m^3^, respectively, in Pudong New Area, Shanghai during the study period.

**Table 1:** Comparison of baseline characteristics between AR (*n* = 105) and the control (*n* = 90)

Characteristics		AR (*n* = 105)	Control (*n* = 90)	*P* value^*^
Age (year)		8.5 ± 2.1	8.9 ± 2.3	0 .322
Gender	Boy	66	57	0.522
	Girl	39	33	
Pregnancy case	Full term	90	78	0.567
	Preterm/Post-term	15	12	
Mode of delivery	Vaginal	42	30	0.208
	cesarean	63	60	
Exclusive breastfeeding within 4 months after birth	Yes	51	59	0.012
	No	54	31	
Parental education	Middle school and below	18	24	0.27
	High school	31	21	
	College and above	56	45	
Number of children per family	1	87	78	0.297
	≥2	18	12	
Parental history of allergic disease	Yes	27	0	<0.01
	No	78	90	
Indoor exposure to smoking	Yes	45	41	0.356
	No	60	49	
Home equipped with air conditioner	Yes	82	74	0.296
	No	23	16	
Family pet (cat or dog)	Yes	15	15	0.369
	No	90	75	
Type of accommodation	Apartment-House	36	36	0.711
	Village	54	42	
	Self-made rural residential	15	12	
Distance from the nearest road (m)	<100	36	29	0.201
	100–300	26	31	
	300–500	18	18	
	>500	25	12	

^*^p value from χ^2^ test.

### PM exposure estimates based on activity-based dynamic

**Figure 1:**
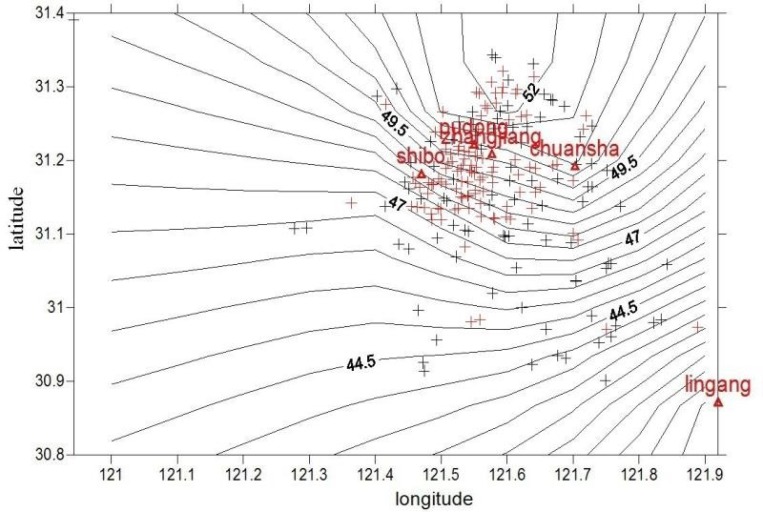
Distribution map of PM_2.5_ concentration near the home addresses of the subjects in Pudong New Area Red + represents the residences of the children with AR while black+ represents the residences of the children in the control group. Pudong, Zhangjiang, Chuansha, Shibo and Lingang represent air quality monitoring stations in Pudong New Area.

**Figure 2:**
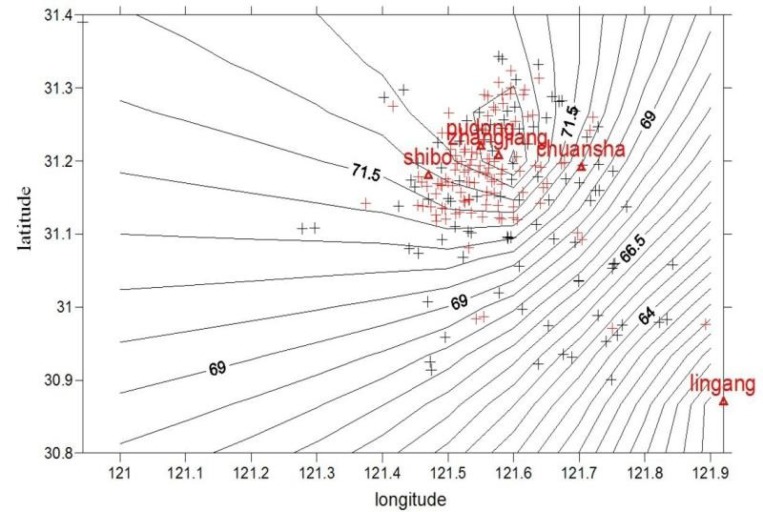
Distribution map of PM_10_ concentration near the home addresses of the subjects with AR in Pudong New Area Red + represents the residences of the children with AR while black + represents the residences of the children in the control group. Pudong, Zhangjiang, Chuansha, Shibo and Lingang represent air quality monitoring stations in Pudong New Area.

Instruction and guidance of allergen control in the daily activities were regularly conducted. The PM concentration distribution near individual home address for the subjects was shown in Figures 1 and 2. The average annual exposure concentrations near personal home address of PM_10_ and PM_2.5_ were 71.9 ± 2.1 μg/m^3^ and 49.5 ± 1.9 μg/m^3^, respectively. PM concentrations were lower in the southern area of Pudong. After calculation by a particle penetration rate coefficient model as well as indoor and outdoor activity time, individual activity-based dynamic exposure to PM_2.5_ and PM_10_ were 43.2 ± 1.6 μg/m^3^ and 62.8 ± 1.8 μg/m^3^ in the children with AR, respectively (Table 2).

**Table 2:** Concentrations of PM applied by Kriging model and estimates based on activity-based dynamic exposure from January 1 to December 31, 2013

Exposure estimates	Subjects	PM_2.5_(µg/m^3^)	PM_10_(µg/m^3^)
Ambient PM	AR	49.5 ± 1.9	71.9 ± 2.1
	Control	47.7 ± 2.9	69.3 ± 3.2
		*p* = 0.037	*p* < 0.001
Activity-based dynamic PM	AR	43.2 ± 1.6	62.8 ± 1.8
	Control	41.6 ± 2.5	60.5 ± 2.8
		*p* = 0.041	*p* < 0.001

PM_2.5_, particulate matter with an aerodynamic diameter less than 2.5 μm; PM_10_, particulate matter with an aerodynamic diameter less than 10 μm.

### DNA methylation around the IFN-γ promoter site in CD4+T cells and IFN-γ expression

**Figure 3:**
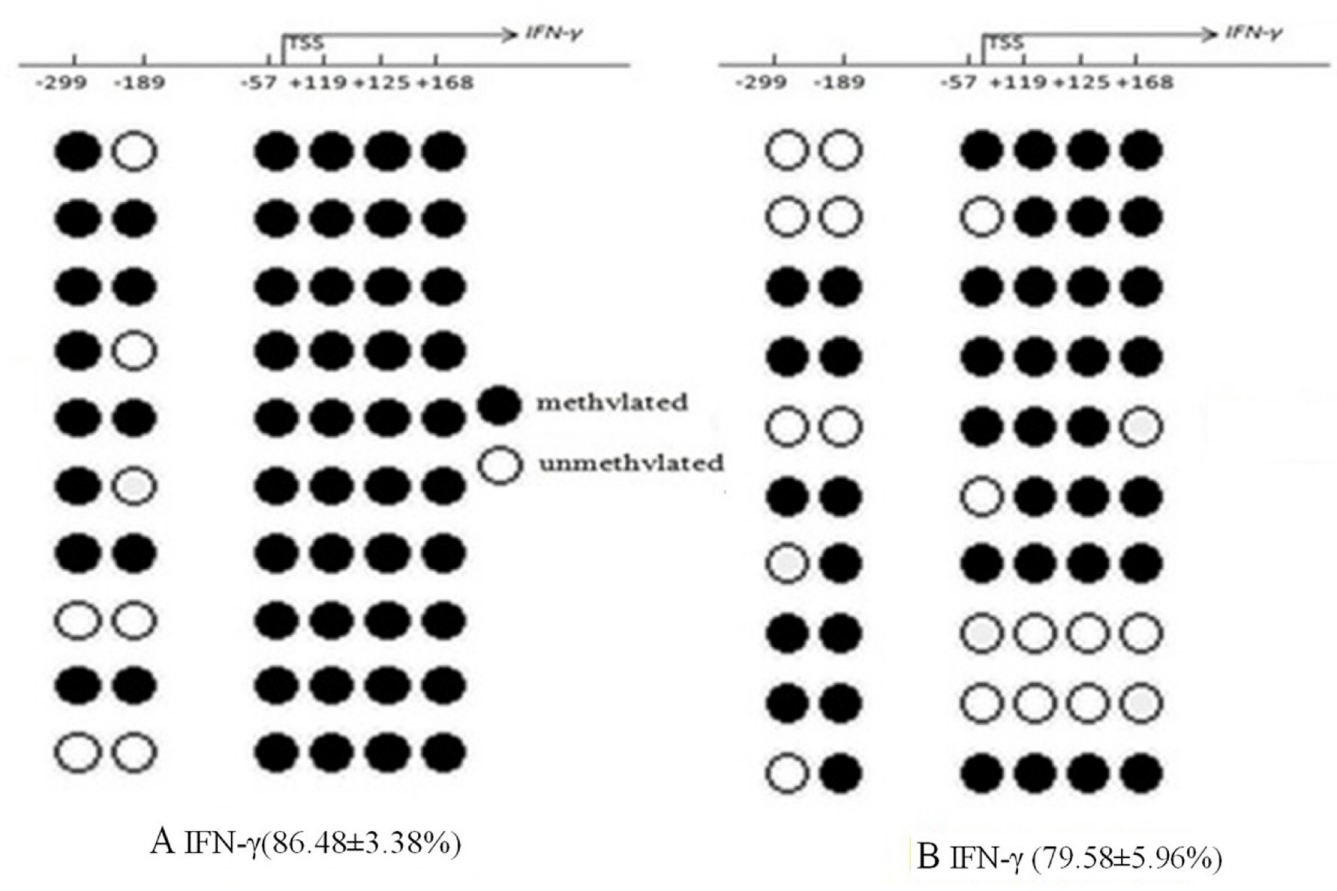
Bisulfite DNA sequencing of six CG loci in two fragments of the IFN-γ promoter region in AR (**A**) and control groups (**B**). Methylated and unmethylated CpG dinucleotides were represented by solid and open circles, respectively. The percentage of methylated CpG dinucleotides was indicated in the parentheses. TSS, transcription start site.

**Figure 4:**
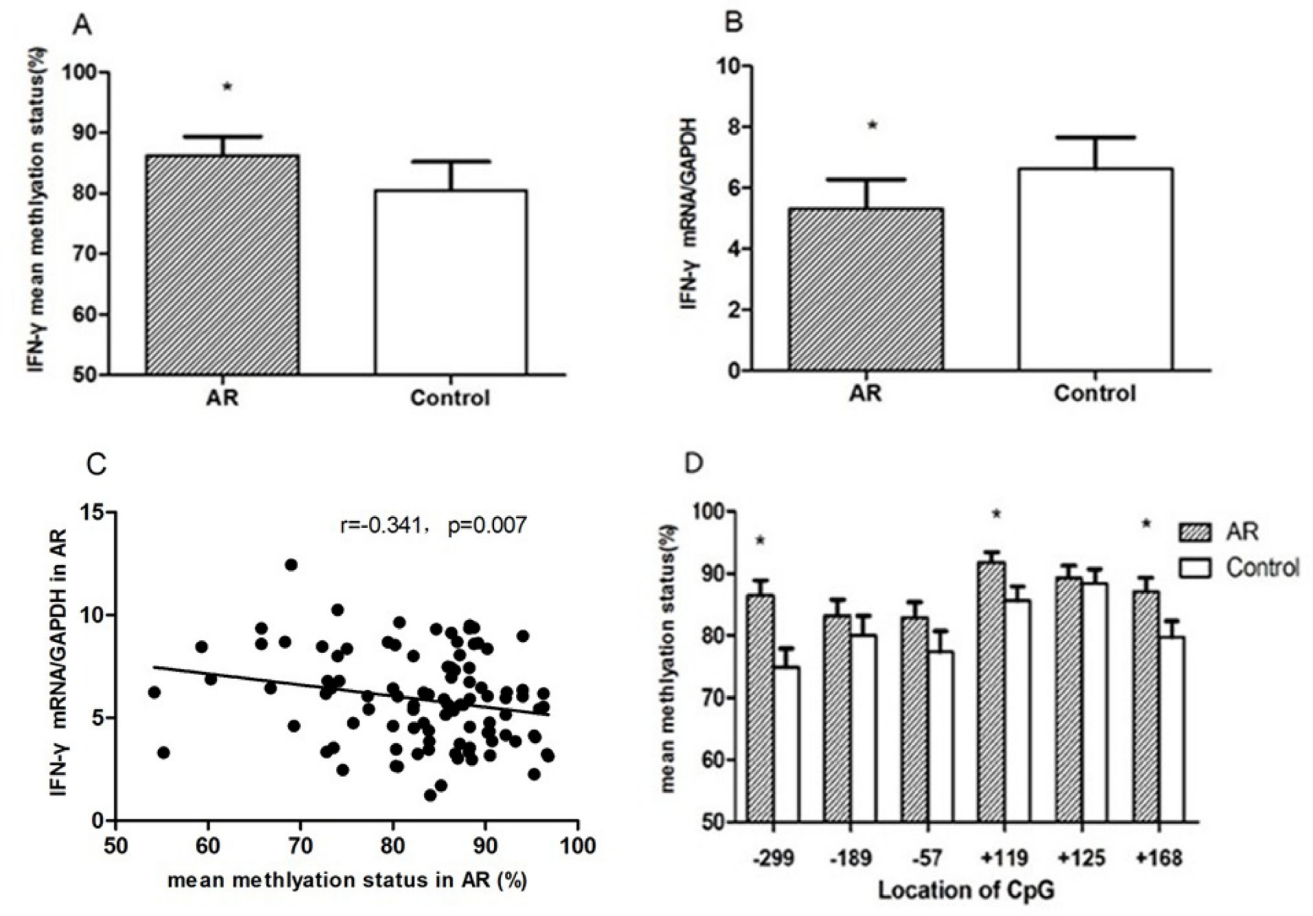
In CD4^+^T cells from AR children and controls mean DNA methylation status of six loci around the IFN-γ promoter site was determined (**A**); relative mRNA levels of IFN-γ were measured by qRT-PCR (**B**). The correlation between IFN-γ mRNA expression and mean methylation level was examined in CD4^+^T cells from children with AR (**C**). The mean methylation status at positions –299, –189, –57, +119, +125 and +168 was compared with the controls, respectively (**D**).^*^p < 0.05.

Compared to the control values, mean methylation status of six CG loci (-299, -189, -57, +119, +125, +168) around the promoter region of IFN-γ in the AR group was significantly higher than those in the control group (86.48 ± 3.38% vs 79.58 ± 5.96%, *p* = 0.042) (Figure 3). IFN-γ mRNA levels in AR CD4^+^T cells were significantly decreased in CD4^+^T cells of the AR group (5.88 ± 2.12 vs 7.55 ± 2.99, *p* = 0.036). IFN-γ transcription levels were negatively correlated with mean DNA methylation level (*r* = –0.341, *p* = 0.007). Among all of the loci, the differences in methylation levels at loci –299, +119, and +168 between AR and control groups were significantly different (*p* = 0.004, *p* = 0.029, *p* = 0.035) (Figure 4).

### DNA methylation around the IL-4 promoter site in CD4+T cells and IL-4 expression

**Figure 5:**
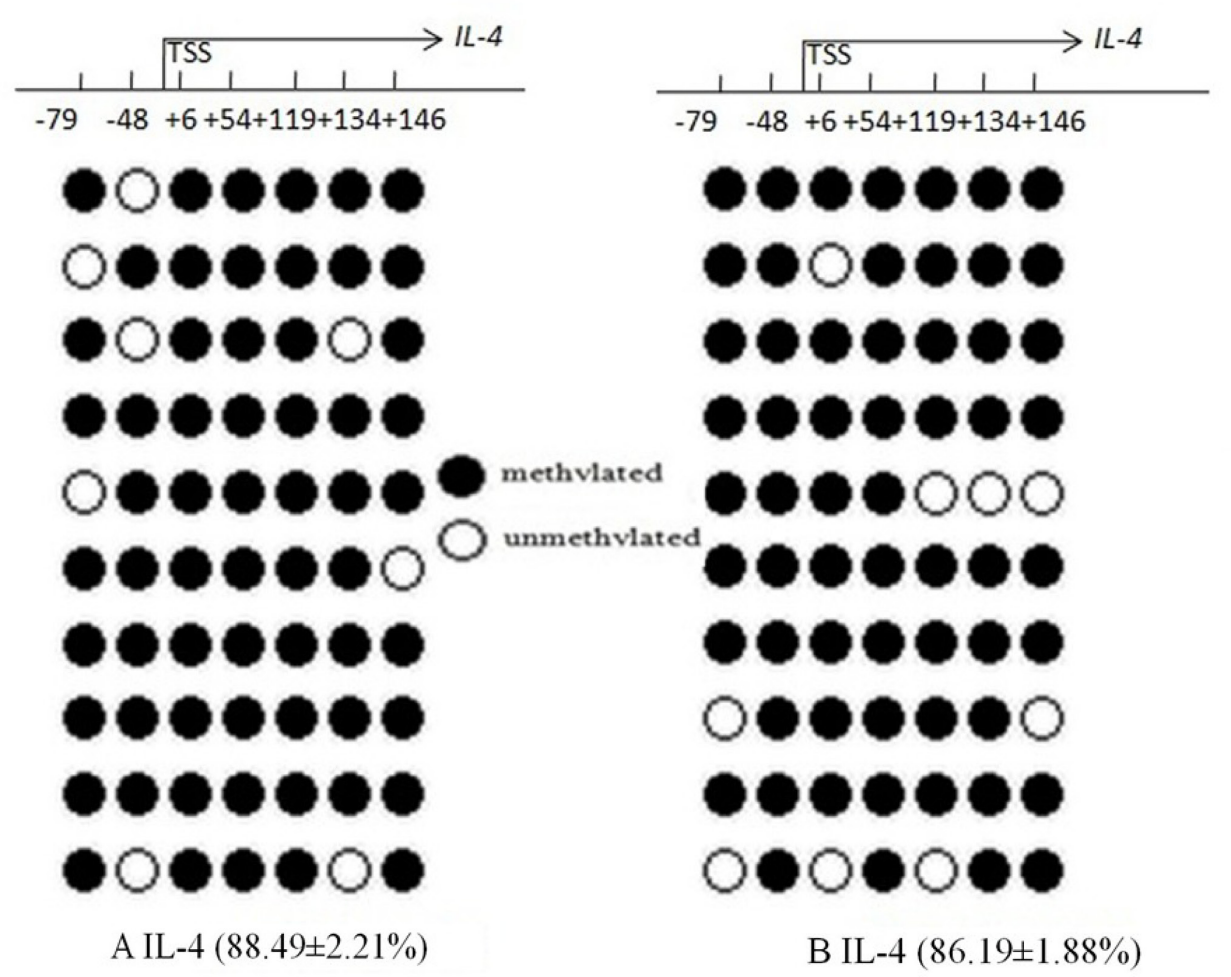
Bisulfite DNA sequencing of seven CG loci in two fragments of the Il-4 promoter region in AR (**A**) and control groups (**B**). Methylated and unmethylated CpG dinucleotides are represented by solid and open circles, respectively. The percentage of methylated CpG dinucleotides is indicated in the parentheses. TSS, transcription start site.

**Figure 6:**
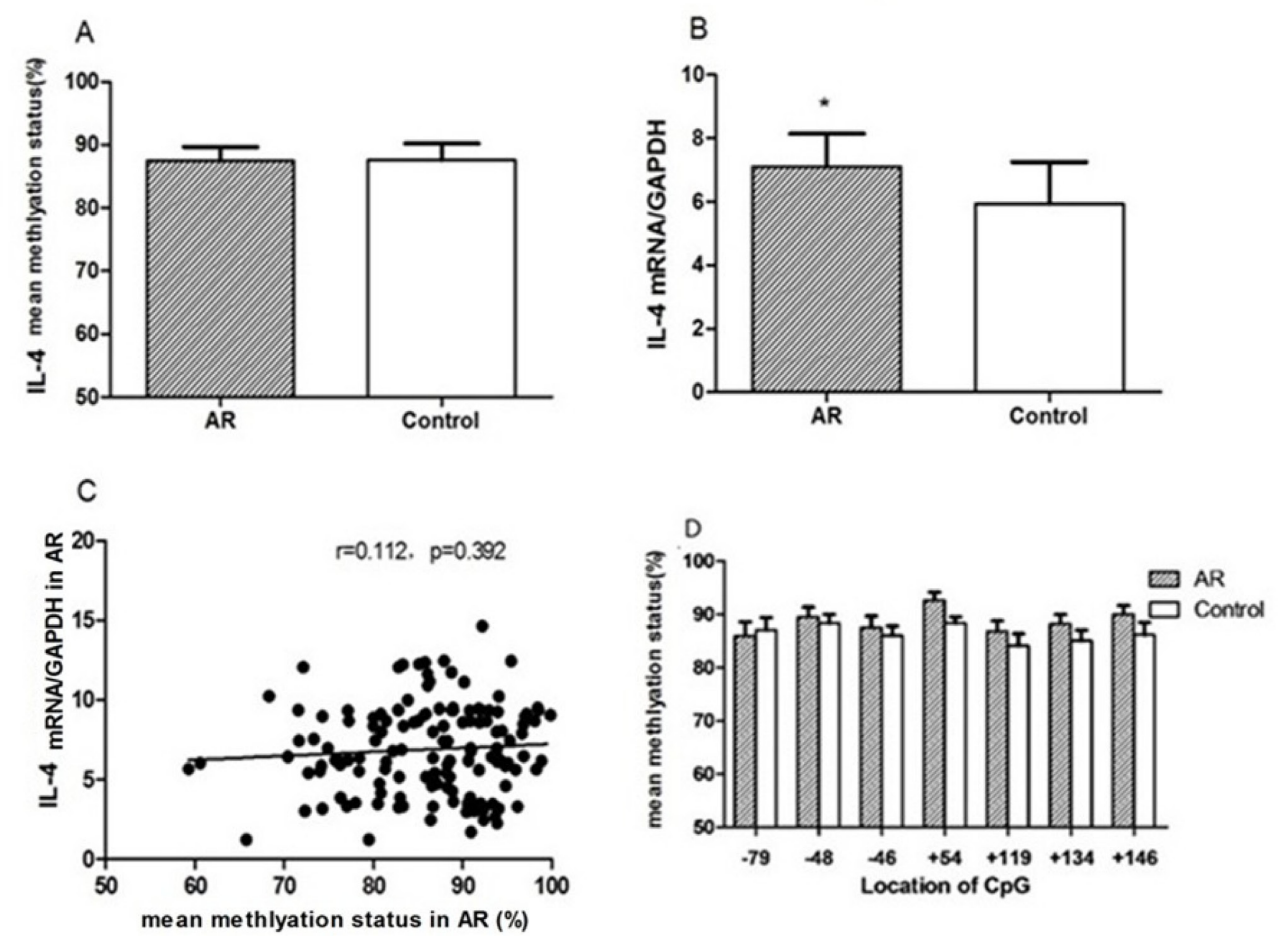
In CD4^+^T cells from children with AR and controls Mean DNA methylation status of seven loci around the *IL-4* promoter site was determined (**A**); relative mRNA levels of IL-4 were measured by qRT-PCR (**B**). The correlation between *IL-4* mRNA expression and mean methylation level was examined in CD4+T cells from children with AR (**C**). The mean methylation status at positions –79, –48, +6, +54, +134, +119, and +146 was compared with the controls, respectively (**D**). ^*^p < 0.05.

The mean methylation status of the seven CG loci (–79, –48, +6, +54, +119, +134, and +146) in the promoter region of IL-4 between the AR and control groups showed no significance (88.49 ± 2.21%vs 86.19 ± 1.88%, *p* = 0.221) (Figure 5). Compared with the control group, IL-4 transcription levels were significantly increased in CD4^+^T cells in the AR group (7.64 ± 2.12 vs 6.50 ± 2.47, *p* = 0.039), and were not correlated with mean DNA methylation level in the AR group (*r* = 0.112, *p* = 0.392) (Figure 6).

### Individual average annual exposure concentrations of PM and the correlation with methylation level

**Figure 7:**
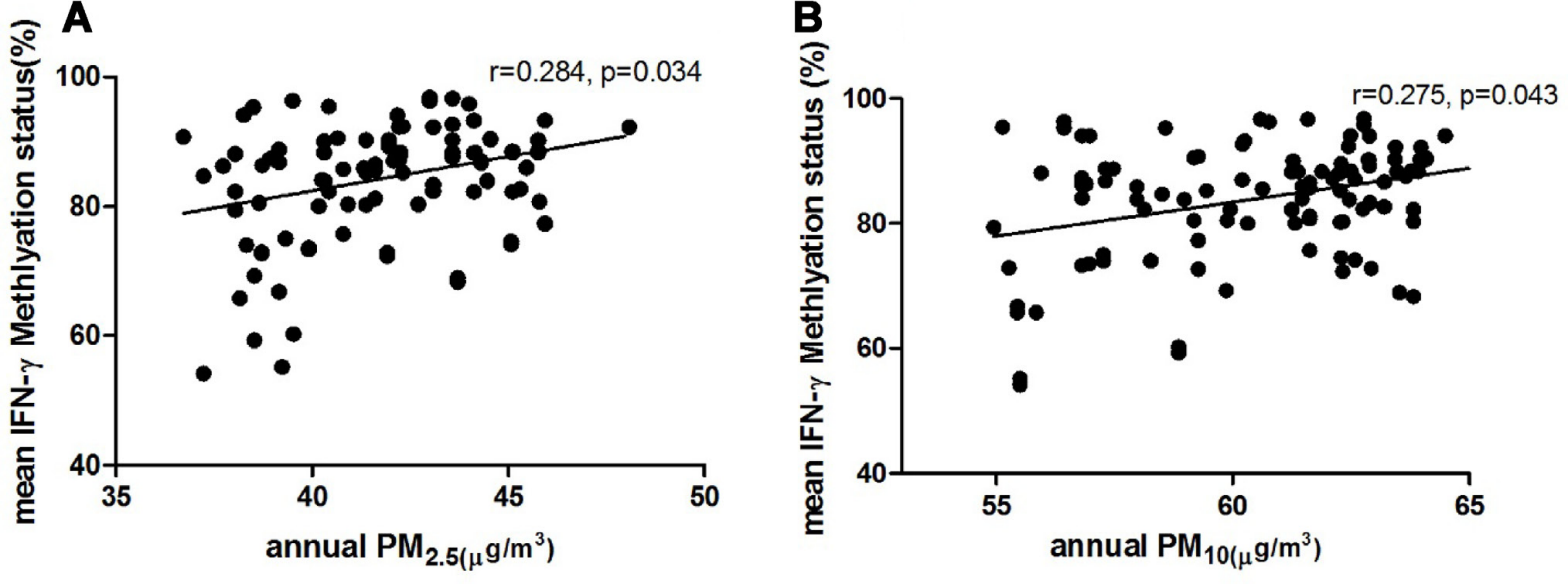
Relationship between individual average annual exposure concentration of PM2.5 (**A**), PM_10_ (**B**) and mean methylation status of the promoter region in IFN-γ.

The average annual exposure concentrations of particulate matter were measured in 105 children with AR who also underwent methylation sequencing. The results showed that annual exposure concentrations of PM_2.5_ and PM_10_ in children were positively correlated with the methylation levels of the promoter region in IFN-γ. (PM_2.5_, *r* = 0.284, *p* = 0.034; PM_10_, *r* = 0.275, *p* = 0.043) (Figure 7).

In multivariate analysis, exposure to PM_2.5_ in AR children was significantly associated with the methylation level of the promoter region in IFN-γ after the adjustment for exclusive breastfeeding within 4 months after birth and parental history of allergic disease, which showed significant difference between the AR and the control (β = 1.441, SE = 0.672, *p* = 0.039). However, PM_10_ was not significantly correlated with the methylation level of the promoter region in IFN-γ (β = 1.025, SE = 0.673, *p* = 0.175) (Table 3).

**Table 3:** β coefficients from multivariate regression model for the methylation level of the promoter region in IFN-γ in children with AR

PM	*β*	SE	*p* value
PM_2.5_ (μg/m^3^)	1.441	0.672	0.039^*^
PM_10_ (μg/m^3^)	1.025	0.673	0.175

The model is adjusted for age, gender, exclusive breastfeeding within 4 months after birth and parental history of allergic disease; ^*^p < 0.05. PM_2.5_, particulate matter with an aerodynamic diameter less than 2.5 μm; PM_10_, particulate matter with an aerodynamic diameter less than 10 μm

## DISCUSSION

AR is a global health challenge with an increasing prevalence over the past few decades. AR in children has a major impact on the quality of daily life including loss of sleep,and productivity, and further undermines the children’s ability to function well during the school day [15]. Environmental factors modulate clinical and immunological phenotype influences on atopic sensitization and allergic disease development in childhood. High PM episodes in Shanghai are widely recognized as one of the major environmental and public health challenges.The fact that high levels of ambient air contaminants enhance the risk of elevated frequency of clinic visits for AR has been reported [16]. IgE mediated allergic reactions to proteins are more severe since PM and some other air pollutants have synergistic effects. The exact mechanism seems to be that air pollutants can affect the immune function by changing the structure of epithelial cells, increasing the sensitivity of specific antigens, and aggravating allergic reaction rather than simple superposition of the effects [17]. However, the detailed mechanisms for the adverse effects of PM inhalation on human health remain unknown. Environmental exposure is believed to induce epigenetic changes, which appears to be an important modifier of disease susceptibility.

In this study, we showed that high methylation at selected CpG sites of the IFN-γ promoter were identified in children with AR for the first time, which may be affected by exposure to PM_2.5_. Therefore, it is very important for early prevention and individualized treatment strategies to evaluate the effects of different risk factors in the environment on pediatric AR.

We used both residential PM estimates by Kriging interpolation model and time-activity-based exposure estimates to ensure the accuracy, given the consideration of individual’s mobility and the fact that spatiotemporal variability of ambient air pollution affects personal exposure estimates, In our study, activity-based dynamic exposure to PM_10_ and PM_2.5_ were 62.8 μg/m^3^ and 43.2 μg/m^3^ in AR children that is nearly 3 -fold the PM annual standards in the USA, which was significantly higher than those in the control group. Exposure estimates derived from individual exposure assessments will provide an opportunity for us to investigate their implications on public health by combining individual exposure estimates with socioeconomic, demographic, and environmental information [18].

Then we compared the methylation status of cytokine genes and assessed the relationship with cytokine mRNA expression between children with AR and the control subjects, identifying significantly IFN-γ high level of methylation in CD4^+^T cells from AR group and altered cytokine expression. In contrast, IL-4 had slightly low level methylation, which was unrelated to high expression of IL-4. In pathogenesis of AR, Th2 differentiation, an important characteristic of chronic allergic inflammation, is prominent that results in T helper cell polarization to Th2. IL-4 and IFN-γ are major cytokines that affect the differentiation of CD4^+^T lymphocytes into Th1 and Th2 lineage. IFN-γ is a highly active and multi-functional cytokine that is released by Th1 cells. IFN-γ promotes Th1 cell differentiation and activates macrophages, resulting in further differentiation of CD4^+^T cells into Th1 cells. IFN-γ can also inhibit the transcription and translation of IL-4 that inhibits Th2 cell differentiation. The differences in the direction of T cell differentiation can be explained by epigenetic variation in signal transmission,.Epigenetics is thought to be a regulator for the transcription of Th genes and cytokine production in the pathogenesis of allergic diseases [19]. Previous studies have shown that the stimulation of CD4^+^T lymphocytes causes considerable increases in the degree of unmethylation *in vitro*^15^ after allergen sensitization and methylation of Th genes was influenced by inhaled environmental exposures in experimental asthma [13]. In addition, a report suggested that PM could enhance aeroallergen sensitization in the respiratory tract, IgE sensitivity to allergens, and susceptibility to respiratory tract infection, which may be caused by upregulated Th2 function [20]. These findings suggested that exposure to PM could modify the function of Th subgroups by epigenetic changes.

To assess the effects on gene expression at transcriptional level, we focused on methylation sites in the promoter regions and the first exon regions of the IFN-γ and IL-4 genes to assess their effects on gene expression at transcriptional level, since the major determinants in the process of T cell differentiation are DNA methylation, DNA-binding proteins, histone acetylation, and transcription factor abundance, which act reciprocally. In the meantime, CpG site is located in the gene promoter region or nucleotide sensitive coding region, which affects gene transcription and can be found in the transcription factor binding sites [13, 21]. Our results showed that the mean methylation level of the IFN-γ gene promoter region in the AR group was significantly higher than that of the control group. CpG^–299^, CpG^+119^ and CpG^+168^ were significantly different, with transcription levels decreasing correspondingly. Previous studies demonstrated that increased DNA methyltransferase (DNMT) amounts could lead to passive methylation of IFN-γ in Th2 polarization [22], and DNA methylation of IFN-γ could inhibit its expression for a long time and play an important role in the cell differentiation process in the polarization status of Th2 cells [23]. Until now, literature on the importance of the total percentage of CpG sites vs. a single CpG site in gene expression remains controversial.

To further evaluate the effect of methylation on the expression of cytokines genes, we calculated the correlations between quantitative cytokine mRNA expressions and the percentage of methylation in both groups. When all subjects were evaluated, a strong negative correlation between mRNA levels of the Th1 cytokine IFN-γ and the percentage of methylation in the AR group was observed. This explanation is consistent with previous reports that DNA methylation in the promoter region of IFN-γ is critical to its gene expression [22, 24]. Other explanation was advocated that demethylation is needed to maintain IL-4 gene hypomethylation in Th1 cells [25]. However, we found no significant change in the unmethylation of IL-4 in the patients, although cytokine mRNA expressions were increased remarkedly. These data suggest that methylation at selected positions has a stronger effect on IFN-γ gene expression in pediatric AR.

Moreover, our findings demonstrate that exposure to activity-based dynamic PM_2.5_ is associated with epigenetic changes of the methylation level in the IFN-γ gene promoter region of the patients’ CD4^+^T cells, which supports the hypothesis that ambient PM exposure may contribute to the persistence of AR symptoms. Several other researches emerged that provide additional evidence with respect to air pollution exposure. In animal models of allergy to Aspergillus through nasal route, we found that inhalation of diesel PM could alter DNA methylation levels of the two important genes IFN-γ and IL-4 in allergic diseases, thereby increasing the secretion of total IgE and resulting in disease onset [7]. Tamar *et al.* assessed141 volunteers and found that exposure to air pollutant PM is significantly related to the methylation status of relevant genes involved in the pathways of asthma pathogenesis [9]. Salam *et al.* [26] first reported that short time exposure to PM_2.5_ is related to low NOS2 and FeNO methylation levels. Our results also suggest that dose-response relationships may exist for PM exposure and IFN-γ. Furthermore, IFN-γ hypermethylation requires DNMT to catalyze and maintain its methylation status, acting on core sequences of the structural gene promoter to inhibit gene expression [22].

There are several limitations in our current study. Firstly, the children’s routine space-time path used in the dynamic exposure estimation is an approximate representation of mobility. An alternative activity time might have been derived if a different spatial and temporal resolution of grids in the model was used, which could affect individuals’ dynamic exposure assessments. Secondly, limited number of related allergic disease genes and CpG sites per gene were studied. The examination of genomic DNA and additional CpG sites may be beneficial to elucidating the mechanism of important epigenetic changes in airway inflammation. Thirdly, without examination of DNA methylation/gene expression in airway cells, it is still unclear how these biological changes in relation to PM_2.5_ in blood samples would play a crucial role in airway inflammation, thus, further functional study would be helpful to validate these results. Lastly, we did not have additional blood tests over one-year time period to periodically demonstrate a “time course” change in the blood, which was due to several reasons. First, ambient PM_2.5_ levels were relatively lower compared to the occupational levels, which may take relatively long time to generate sizable adverse health effects. Some epigenetic impacts from chemical exposure may take one year or more to occur as well. Therefore, the study was designed to collect one time blood samples once at the end to investigate cumulative effects over one year time period. Secondly, feasibility of blood sample collection, especially in school children in our case, was very limited. Nonetheless, the most important strength of this study was its ability to examine the effect of long-term PM exposure on CpG methylation patterns of IL-4 and IFN-γ promoter genes in CD4^+^ cells in pediatric AR. To the best of our knowledge, this is one of the first studies showing the contributions of DNA methylation on allergic inflammation in PM mediated phenotype expression of AR.

## CONCLUSIONS

Our results demonstrate the critical role of DNA methylation of CD4^+^T cells in decreasing IFN-γ gene mRNA expression in children with AR. Promoter region methylation in IFN-γ gene varied with exposure to ambient PM_2.5_ concerning individual activity-based dynamic exposure. These findings suggest the effect of PM_2.5_ mediated epigenetic modification may lead to increased susceptibility for the development of allergic rhinitis. How these epigenetic changes contribute to or modify the effects of air pollution on pediatric AR deserves further investigation, and may provide foundation for possible prevention of AR.

## MATERIALS AND METHODS

### Study subjects

According to the prevalence of AR in China, a sample size of 200(each group about 100) was sufficient to detect the odds ratio of 3.2 in the high PM exposure population with power of 80% at the 5% level of significance. A total of 10 5pediatric patients (66 boys and 39 girls) with AR were prospectively recruited from Department of Otorhinolaryngology at Shanghai Children’s Medical Center from January 1, 2013 to December 31, 2013. In addition, 90 controls who were healthy volunteers from the Department of Children Health Care in our hospital were also recruited from the same region (Shanghai and the surrounding region) with long-term residence in the locality that matched the AR group. All study subjects were aged 6–12 years. This study was approved by the Ethics Committee of Shanghai Children’s Medical Center. Based on the principle of informed consent, all parents of the subjects were fully informed and signed consent forms.

Patients were diagnosed with AR if they m*et al*l three of the following criteria from the ARIA (Allergic Rhinitis and its Impact on Asthma, 2008) [21], with recurrence of symptoms over a year: 1) persistent symptoms of water-like tears, nasal itching, congestion, sneezing, and other symptoms; the symptoms lasted for more than 1 hour every day and could be accompanied by eye symptoms such as itchy eyes and conjunctival congestion; 2) pale nasal mucosa, edema, and nasal water-like secretions were the common signs on physical examination; 3) one or more positive specific inhalant allergens were found (>0.35 kU/ml) (SIgE, Western blotting) (Allergy Screen™ human serum specific IgE allergen detection kit) in detection of specific IgE in serum, in which tree powder, ragweed, artemisiaargyi, house dust mite, house dust, cat/dog hair, cockroach, penicillium notatum, aspergillus fumigatus, humulus scandens were included. In this study, childrenaged 6–12 years were recruited. The control group whose total serum IgE and SIgE were negative underwent routine physical examinations, with no diagnosis of atopic diseases (such as AR, asthma and eczema).

The exclusive criteria in the AR group were: 1) a history of one-year living outside of Shanghai; 2) recent major events such as newly decorated house, relocation, transfer, separation from caring person, and hospitalization; 3) school outside the same area as residence; 4) combination of asthma with eczema; 5) autoimmune diseases, congenital airway, heart diseases, or malignant tumors. The cases who met any of these criteria were excluded.

### Environmental data

From January 1st, 2013 to December 31st, daily PM_2.5_ and PM_10_ data from five fixed-site stations in Pudong New Area, including Chuansha, Shibo, Pudong, Zhangjiang and Lingang, which are affiliated by Shanghai Center for Urban Environmental Meteorology under China National Quality Control, were obtained.

### Individual environment assessment questionnaires

Home address, history of allergic disease, exclusive breastfeeding in the first 4 months, parental educational level, and number of children in the household, family pets, secondhand smoke exposure (home exposure to smoking parents or any other smoking person), housing type and distance from the nearest street, were included. The gestational time of 37–41 weeks was defined as full term pregnancy, while <37 weeks and >42 weeks were defined as preterm and post-term births, respectively. Parents with AR, allergic conjunctivitis, asthma and eczema were defined as having a family history of allergic diseases. The locations where an individual visited, as well as the timing, duration, sequencing and the types of activities, were recorded daily.

### Dynamic personal PM exposure assessment in children

Annual activity-based dynamic exposure to PM was calculated by using a particles penetration rate coefficient model in Shanghai as well as indoor and outdoor activity time [4, 14]. The floor of the apartments, air condition, ventilation habit, indoor temperature and other factors were considered when the indoor PM penetration rate was built. Because the people’s ventilation habits in shanghai were generally similar, the difference of indoor PM penetration rate was minimal among different apartments. However, the penetration rated differed between different seasons. Therefore, we calculated the average indoor PM penetration by season in our study.

PM_I/O (average PM_2.5_ indoor penetration coefficient) = (3 * 0.86 + 0.81)/4 = 0.85 (integrated indoor and outdoor penetration coefficients for different seasons)

Indoor rate (average indoor activity time) = (3 * inrate_summer+inrate_winter)/4 (proportion of indoor activities for different seasons)

Exposure = Indoor_rate * 0.85 * PM_2.5_ + (1-indoor_rate)*PM_2.5_ (individual exposure calculation)

The specific value of penetration coefficient of PM_10_ in Shanghai was not obtained at that time, so the same value from PM_2.5_ was used for PM_10_.

### Laboratory methods

#### Sample collection and CD4+ T cell isolation

Peripheral venous blood was collected and centrifugal column blood genomic DNA extraction kit (QIAamp DNA Micro kit, Qiagen) was used for venous blood genomic DNA extraction. Nucleic acid detection instrument (NANODROP 2000) was used to assess DNA quantity and purity. Isolation of peripheral blood mononuclear cells (PBMCs) and flow cytometry sorting of CD4^+^T lymphocytes were performed. Cell purity was greater than 98%, with a count above 1 × 10^6^ after sorting.

The target areas for IFN-γ (NG_015840.1) and IL-4 (NG_023252.1) were chosen based on previous studies of epigenetic regulation following inhaled environmental exposures [13, 21]. Data was downloaded from UCSC website (http://genome.ucsc.edu/, GRCh37/hg19), including 2kb upstream of the 5′end and a full-length gene sequence. CpG islands in the target sequence were predicted with the related software from http://emboss.bioinformatics.nl/cgi-bin/emboss/cpgplot. Premier 3 software was used for the analysis. Primers were selected and the transcription start site was defined as +1 (Table 4).

**Table 4:** IFN-γ and IL-4 fragment primers for bisulfite sequencing

Gene	Primer^a^	PCR fragment lengths(bp)	Sequence(5′-3′)
*IFN-γ*	Sequencing F1(CpG^–299^, CpG^–189^)	194	GTAAATGATTAATGTGTTTTGTGAATGAAG
(NG_015840.1)	Sequencing R1(CpG^–299^, CpG^–189^)		TAAAATTTCCTTTAAACTCCTTAAATCC
	Sequencing F2(CpG^–57^, CpG^+119^, CpG^+125^, CpG^+168^)	391	GGATTTAAGGAGTTTAAAGGAAATTTTA
	Sequencing R2(CpG^–57^, CpG^+119^, CpG^+125^, CpG^+168^)		ACATATAAATCCTAACAATAACAACCAAA
*IL-4* (NG_023252.1)	Sequencing F (CpG^–79^, CpG^–48^, CpG^+6^, CpG^+54^,CpG^+119^, CpG^+134^,CpG^+146^)	306	TTGTGAGGTTGTTTAAAGTTTTGATG
	Sequencing R(CpG^–79^, CpG^–48^, CpG^+6^, CpG^+54^,CpG^+119^, CpG^+134^,CpG^+146^)		CTAATTAACCCCAAATAACTAACAATCTAA

^a^Site based on previous studies of *IFN-*γ and *IL-4*;F, forward primer; R, reverse primer.

### Bisulfite treatment and PCR amplification of bisulfite converted DNA

Methylation treatment was performed to convert unmethylated cytokines to uracil and leaving methylated cytokines unchanged with EZ DNA Methylation-Gold Kit (Zymo Research). The polymerase chain reaction (PCR) amplification was performed for DNA samples with 3 fragment primers of the target genes. The total reaction volume was 15 μl, including 1.5 μl of 1x HotStarTaq buffer, 1 μl of Mg^2+^ (25 mM), 0.5 μl of dNTP (10 mM), 0.5 μl of each forward and reverse primers (10 μM), 0.15 μl of HotStarTaq polymerase (5 U/μl), 1 μl of template DNA (100 ng), and 2 μl of ddH_2_O. Reaction conditions: pre-denaturation at 95°C for 15 min, denaturation at 94°C for 30 s, annealing at 55°C for 30 s, and extension at 72°C for 1 min, for a total of 40 cycles; final extension at 72°C for 5 min. PCR products were purified with Qiagen’s QIA quick Purification Kit (Catalog number. 28104) and stored at –20°C.

### Cloning and sequencing

**Figure 8:**
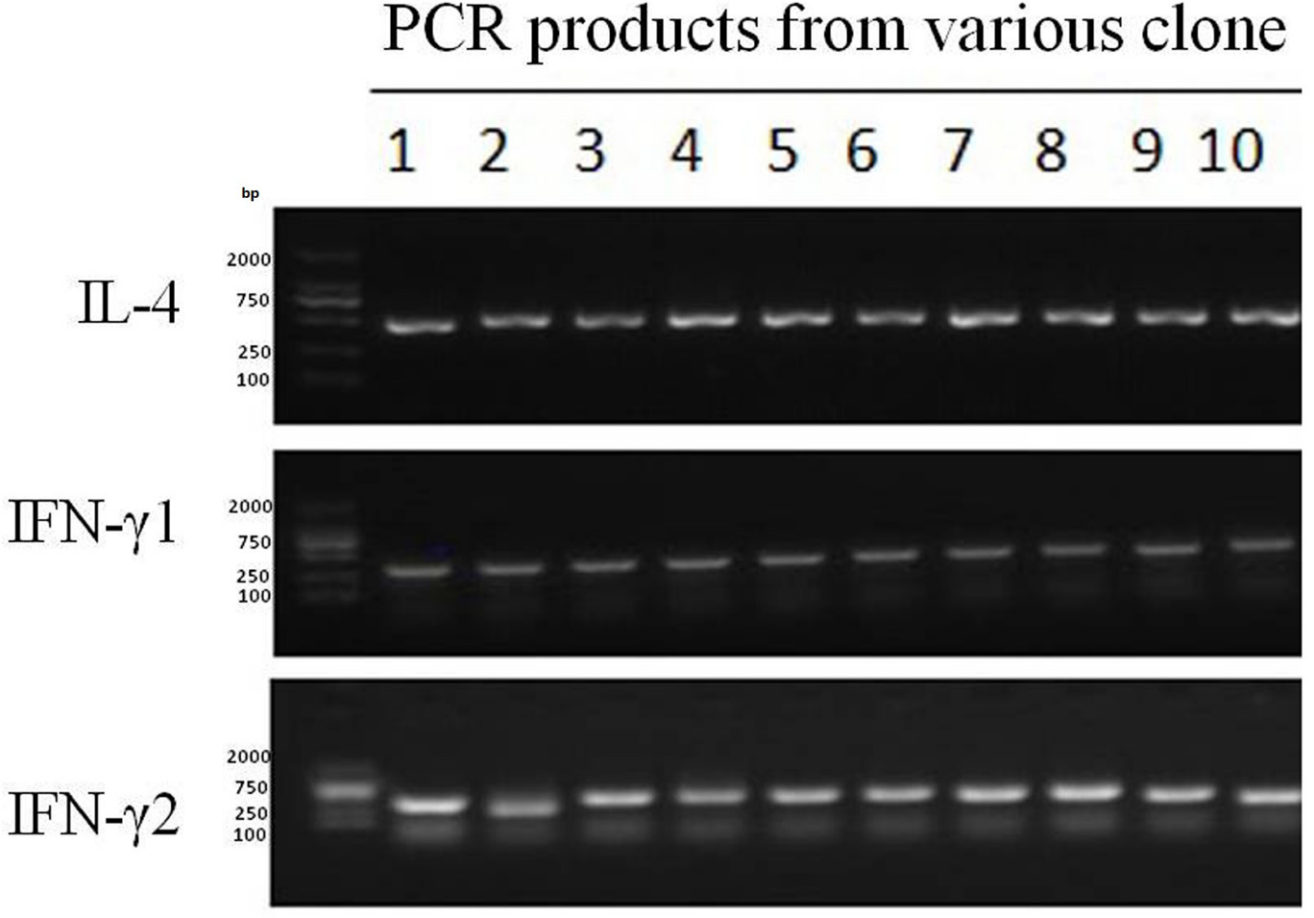
PCR products of IFN-γ fragments 1 and 2, and IL-4 fragment in various clones, as detected by electrophoresis

Purified PCR products were cloned into pMD18-T simple vector with TAKARA T carrier link Kit (D101A) and plated onto KAN-X-GAL plates for blue-white screening. Positive colonies were inculated into LB-Amp+ tubes (volume ratio, 1000:1), cultured at 37°C with 180rpm and shaken for more than 3 hours. PCR identification was performed using specific PCR primers. Identification of PCR products was performed by 1.5% agarose gel electrophoresis (Figure 8). To address the heterogeneity of DNA methylation patterns, 10 or more clones/alleles were sequenced per patient for each of the targeted loci.

Bacterial suspensions of 10 positive clones identified by PCR were randomly selected in each sample and sequenced. Methylation ratio of CG loci in the 10 clones was assessed by template sequence alignment using the Sequencher software.

### RNA isolation and real-time quantitative RT-PCR (qRT- PCR)

Total RNA was extracted from CD4^+^T lymphocyte by Roche Tripure RNA isolation kit, and RNA concentration and purity were determined via a NanoDrop spectrophotometer. Prime Script™ RT reagent Kit (TakaRa) was used for reverse transcription. Full-length sequences of related cDNAs were obtained from GenBank of NCBI. PCR primer design was performed using the online primer design software Primer3 (http://fredo.wi.mit.edu/) according to the primer design principle of fluorescent quantitative PCR primers. The primers were synthetized by BGI Shanghai Biotechnology Company limited. GAPDH was used as reference. Primer sequences are provided in Table 5.

**Table 5:** Primer sequences for qRT-PCR

Gene	Primer	Sequence (5′–3′)
*IFN-γ*(NM_000619.2)	F	AACGAGATGACTTCGAAAAGCTGA
	R	TTCAGCCATCACTTGGATGAGTTC
*IL-4*(NM_000589)	F	GTGCTCCGGCAGTTCTACAGC
	R	GGTTGGCTTCCTTCACAGGACA
*GAPDH*(NM_002046)	F	CGGAGTCAACGGATTTGGTCGTAT
	R	AGCCTTCTCCATGGTGGTGAAGAC

F, forward primer; R, reverse primer.

Primers were dissolved in ddH_2_O for 100 μM stock solution and 2 μl and 18 μl of ddH_2_O were mixed to yield 10 μM working solution. Real-time PCR was performed with SYBR^®^Premix Ex Taq™. Real-time reaction mixtures were prepared (on ice) in 20 μl total volume comprising 10 μl SYBR^®^Premix Ex Taq™ II (2×), 0.4 μl each forward and reverse PCR Primers (10 μM), 0.4 μl ROX Reference Dye II (50×), 2 μl cDNA template (100 ng), and 6.8 μl ddH_2_O. Real-time PCR amplification was performed with the two-step method: pre-degeneration, 95°C for 30 s; PCR reaction, 95°C, 3 s; 60°C, 25 s two-step amplification for 40 cycles. In addition, dissociation curve analysis was performed at 95°C for 15 s, 60°C for 1 min, and 95°C for 15 s. –ΔCT value = CT value of target gene – CT value of housekeeping gene, and was used for data analysis. The relative expression of mRNA was derived as 2^–ΔCT^×100%. Three replicates were used in the amplification reaction, and standard deviation was calculated.

### Statistical analysis

Measurement data were presented as mean ± standard deviation (SD), and enumeration data as number of cases (%) that were normally distributed. The differences between groups for continuous variables were compared by *t*-test to determine the significance of PM exposure, methylation status and mRNA expression. The categorical variables were examined by chi-square test. Individual exposure concentration of PM was assessed via the PM station near the participants’ residence address by the Kriging interpolation analysis using Surfer 10 (Golden Software, Inc.), which was calculated based on the activity time indoor and outdoor and the children’s address concentrations. Spearman rank correlation was used to analyze the associations of the level of individual exposure to pollutants and biological indexes. Multiple linear regression analysis was performed to assess the relationship between the level of exposure and methylation. *p* < 0.05 was considered statistically significant. SPSS statistical software package for Windows version 17.0.1(SPSS Inc., Chicago, IL, USA) was used for all statistical analyses.

## Abbreviations

AR: allergic rhinitis
PM: particulate matters
Th1: type I helper T cells
Th2: type II helper T cells
PM_2.5_: particulate matter with an aerodynamic less than 2.5 μm
PM_10_: particulate matter with an aerodynamic diameter less than 10 μm
